# ACE as a Mechanosensor to Shear Stress Influences the Control of Its Own Regulation via Phosphorylation of Cytoplasmic Ser^1270^

**DOI:** 10.1371/journal.pone.0022803

**Published:** 2011-08-25

**Authors:** Valerio Garrone Barauna, Luciene Cristina Gastalho Campos, Ayumi Aurea Miyakawa, Jose Eduardo Krieger

**Affiliations:** Laboratory of Genetics and Molecular Cardiology, Heart Institute (InCor), University of Sao Paulo Medical School, Sao Paulo, Sao Paulo, Brazil; Consejo Superior de Investigaciones Cientificas, Spain

## Abstract

**Objectives:**

We tested whether angiotensin converting enzyme (ACE) and phosphorylation of Ser^1270^ are involved in shear-stress (SS)-induced downregulation of the enzyme.

**Methods and Results:**

Western blotting analysis showed that SS (18 h, 15 dyn/cm^2^) decreases ACE expression and phosphorylation as well as p-JNK inhibition in human primary endothelial cells (EC). CHO cells expressing wild-type ACE (wt-ACE) also displayed SS-induced decrease in ACE and p-JNK. Moreover, SS decreased ACE promoter activity in wt-ACE, but had no effect in wild type CHO or CHO expressing ACE without either the extra- or the intracellular domains, and decreased less in CHO expressing a mutated ACE at Ser^1270^ compared to wt-ACE (13 vs. 40%, respectively). The JNK inhibitor (SP600125, 18 h), in absence of SS, also decreased ACE promoter activity in wt-ACE. Finally, SS-induced inhibition of ACE expression and phosphorylation in EC was counteracted by simultaneous exposure to an ACE inhibitor.

**Conclusions:**

ACE displays a key role on its own downregulation in response to SS. This response requires both the extra- and the intracellular domains and ACE Ser^1270^, consistent with the idea that the extracellular domain behaves as a mechanosensor while the cytoplasmic domain elicits the downstream intracellular signaling by phosphorylation on Ser^1270^.

## Introduction

Angiotensin-converting enzyme (ACE) is a key component of the renin-angiotensin system, which regulates blood pressure, electrolyte balance and fluid homeostasis [1]–[2]. ACE is a transmembrane protein expressed on the surface of many cell types that can process different peptides through two active catalytic sites in the extracellular domain, including angiotensin I to generate the vasoconstrictor peptide angiotensin II and the degradation of bradykinin [3]. ACE is expressed mainly in endothelial cells (EC), which is highly exposed and sensitive to hemodynamic stimuli such as shear stress (SS) that participates in the short- and long term control of vascular structure and function [4]. The mechanotransduction involved in these processes is not fully elucidated and several mechanosensors have been described. They are usually transmembrane proteins, which can sense the extracellular hemodynamic stimulus and convert it in chemical intracellular response [5].

Recently, it has been demonstrated that the cytoplasmic tail of ACE is phosphorylated on Ser^1270^ (p-Ser^1270^) by ACE inhibitors (ACEi), which then triggers intracellular signaling cascade that leads to increase ACE expression [6]. The binding of ACEi to ACE induces p-Ser^1270^ mediated by casein kinase 2 activating MKK7 and JNK. Then, phosphorylated c-jun activates AP-1 transcription factor and increase ACE expression [7],[8],[9].

We have described that SS suppresses ACE gene expression and activity *in vitro* using a cell system and *in vivo* in the rat aorta [10]. The classical shear stress responsive element (SSRE) is present in the ACE promoter but it is not functional. Indeed, we provided evidence that SS-induced ACE downregulation requires the integrity of two alternative cis-acting elements, Barbie and GAGA-boxes [11]. In addition, we and others have showed that although nitric oxide (NO), a potent vasodilator, antioxidant and anti-inflamatory mediator synthesized and released by SS, influences basal ACE levels in the static conditions [12], it is not associated with the SS-induced ACE gene suppression [13].

Considering that ACE is a transmembrane protein mainly expressed in EC, we now investigated whether the extra- and intracellular domains of ACE and the phosphorylation of a cytoplasmic residue are involved in the mechanotransduction of ACE regulation by SS. The results obtained suggest that phosphorylation of ACE and downstream JNK inactivation participate in this process in primary EC. Using the CHO cell model system, we provided additional evidence that both the extra- and the intracellular ACE domains and Ser^1270^ appear to be required for sensing and eliciting the SS-induced ACE downregulation.

## Results

### SS-induced down regulation of ACE and intracellular cell signaling in SVEC

**Figure 1:**
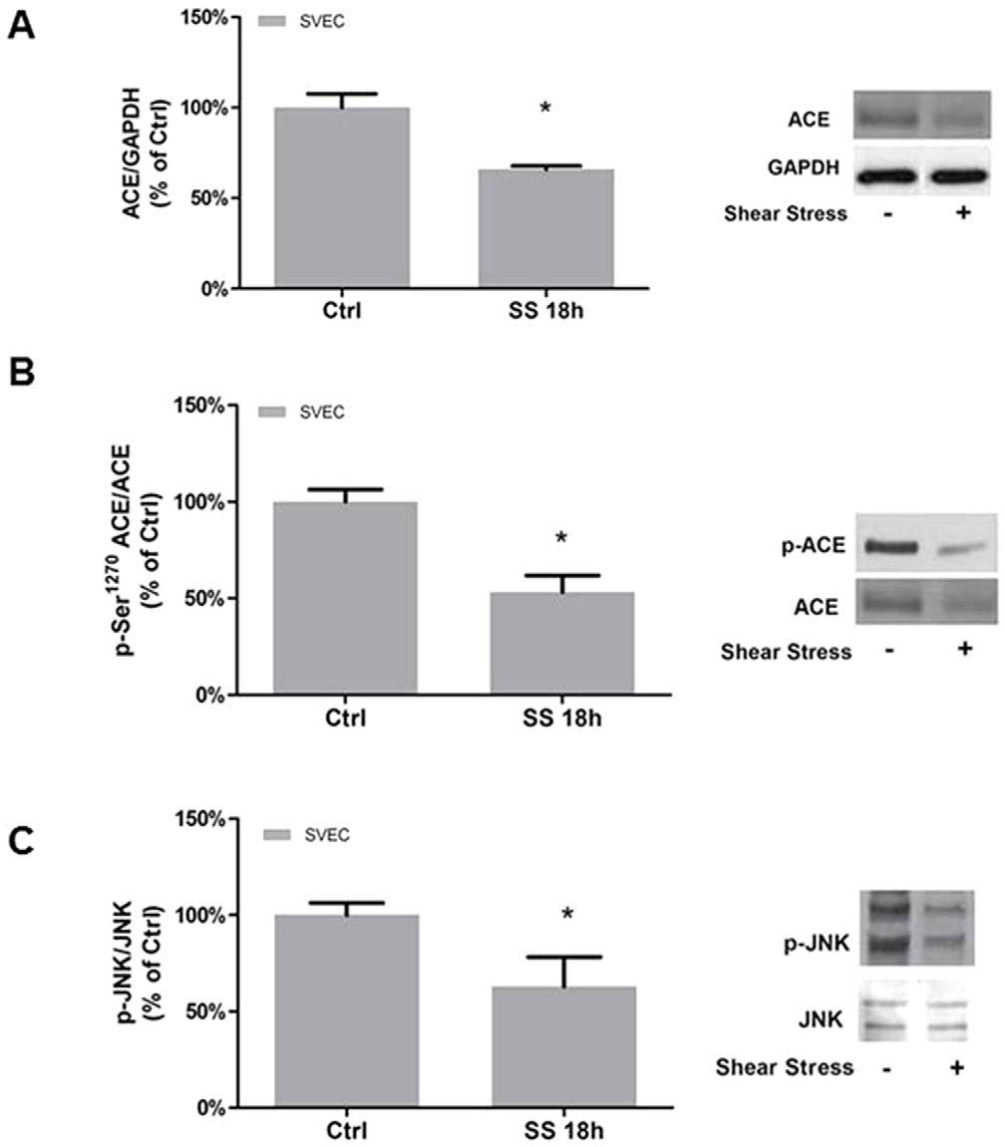
Shear stress diminishes ACE expression and signaling in Human Safenous Vein Endothelial Cells (SVEC). (A) ACE protein expression downregulation, (B) ACE phosphorylation on Ser^1270^, and (C) p-JNK in response to laminar shear stress. SVEC were exposed to 18 h of laminar shear stress (15 dyne/cm^2^; SS 18 h). Each bar represents mean ± SEM of 5 separate experiments. *p<0.05 vs static control (CTRL).

SS (18 h, 15 dyn/cm^2^) in human SVEC is associated with a decrease in ACE protein expression (Figure 1 A), similarly to what we had previously observed in the rat aorta for both activity and mRNA levels while nitric oxide synthase activity increased [10]. Noteworthy, ACE phosphorylation on Ser^1270^ also decreased in response to SS, so that the ratio p-ACE/ACE diminished 47% (p<0.05) indicating that SS was accompanied by reduction in the percentage of p-ACE (Figure 1B).

JNK, one of the downstream signaling pathways influenced by ACE phosphorylation on Ser^1270^ ACE inhibitors bind to the molecule [6]–[9], was also modulated. Upon SS (18 h, 15 dyn/cm^2^), p-JNK significantly decreased by 37% (Figure 1C).

### Construction of CHO cells lineages expressing wild-type and mutated ACE

**Figure 2:**
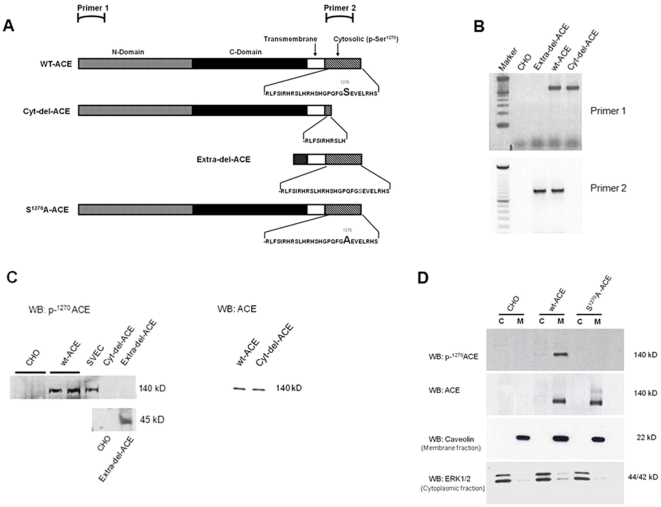
ACE mutants used to dissect the SS-induced response in CHO cells. (A) Schematic diagram of wt-ACE, Cyt-del-ACE, Extra-del-ACE, and S^1270^A-ACE. (B) Analysis of ACE constructs expression in the cell lineages by PCR using specific primers to extracellular (Primer 1) and intracellular (Primer 2) domain of ACE. (C) Representative western blots using antibody against ACE Ser^1270^ phosphorylation or total ACE expression (by Dr Sergei M Danilov). 10 ug of protein was loaded in the gel for each sample. (D). Representative western blots to demonstrate S^1270^A-ACE mutant localized on cell membrane fraction (C for cytoplasmic fraction and M for membrane bound fractions).

Considering the phenomenon described above in primary human endothelial cells, we used CHO cells that do not express the classical components of the renin angiotensin system to directly dissect the role of ACE domains in the SS-induced response and its influence on triggering changes in gene expression. CHO were genetically modified to permanently express the wild type or mutated ACE molecules lacking the extracellular (Extra-del-ACE) or the cytoplasmic (Cyt-del-ACE) domains and carrying a point mutation at the cytoplasmic domain residue 1270 (S^1270^A-ACE) (Figure 2A). Each cell lineage was confirmed by both ACE mRNA and protein expression and displayed the proper membrane bound localization (Figure 2B, 2C and 2D).

For gene expression analysis, PCR experiments were performed using 2 different primer pairs: Primer 1, specific for ACE extracellular domain, and Primer 2 specific for ACE cytoplasmic domain. Note that Prime 1 detected wt-ACE and Cyt-del-ACE but did not detect Extra-del-ACE cells (Figure 2B), accordingly Primer 2 amplified wt-ACE and Extra-del-ACE but not Cyt-del-ACE cells (Figure 2B). Western blot analysis using an ACE antibody confirmed that wild-type CHO do not express ACE while the protein is detected in wt-ACE (∼140 Kd), similar to the signal from primary culture of saphenous vein endothelial cells, and Extra-del-ACE (∼45 Kd) (Figure 2C). In addition, an ACE activity assay showed that the Cyt-del-ACE cell lineage displayed some level of ACE activity while it was completed abrogated in the Extra-del-ACE that lacks the catalytic domain compared with wt-ACE (wt-ACE: 4587±699 uF.min^−1^.mg^−1^; Cyt-del-ACE: 2815±345 uF.min^−1^.mg^−1^; S^1270^A-ACE: 3782±18 uF.min^−1^.mg^−1^; Extra-del-ACE: 192±18 uF.min^−1^.mg^−1^). Using the p-Ser^1270^ ACE antibody, it was demonstrated that wild-type CHO cells and S^1270^A-ACE fail to express phosphorylated ACE in the membrane fraction while the signal is clearly observed in the membrane fraction of the wt-ACE (Figure 2D).

### SS-induced ACE downregulation requires ACE mechanotransduction

**Figure 3:**
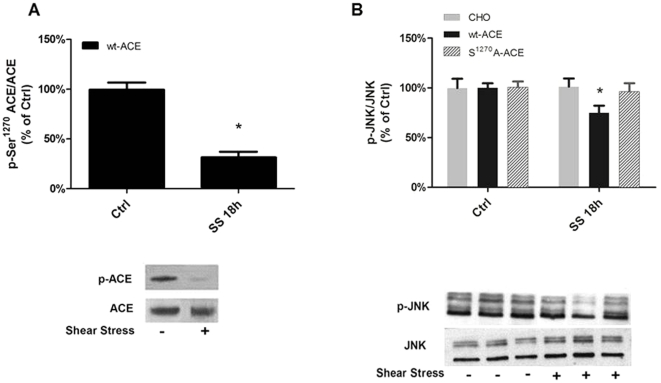
CHO cells expressing ACE recapitulate the behavior of endothelial cells. ACE phosphorylation on Ser^1270^ and p-JNK is diminuished in CHO cells expressing wild-type ACE (wt-ACE) submitted do laminar shear stress. (A) ACE phosphorylation on Ser^1270^ in response to laminar shear stress in wt-ACE cells. (B) p-JNK in CHO, wt-ACE and S^1270^A-ACE cells in response to laminar shear stress. Cells were exposed to 18 h of laminar shear stress (15 dyne/cm^2^; SS 18 h). Each bar represents mean ± SEM of 4 to 6 separate experiments. *p<0.05 vs static control (CTRL).

Similarly to the response in human EC (Figure 1), SS-induced decrease in p-Ser^1270^/ACE ratio (67%) was observed (Figure 3A). This response was accompanied by a significant decrease in p-JNK in wt-ACE cells while it failed to occur in mock transfected CHO cells or in S^1270^A-ACE exposed to the same stimulus (Figure 3B). This is a direct demonstration of the role of ACE phosphorylation in JNK diminished activity by SS.

This observation is consistent with the idea that SS-mediated p-JNK requires ACE and it raises the question about the relative importance of each of the ACE domains, whether the extra-, the intra-, which harbours Ser^1270^, or both, may indeed be involved with the SS-induced response.

Since we had previously demonstrated that a 1273 bp of the rat ACE promoter suffices to display the SS-induced suppression of promoter activity [10], this reporter gene assay was used as a functional downstream surrogate of the SS-induced response in CHO cells to dissect the role of the different ACE domains for this response and the potential link with the JNK pathway.

**Figure 4:**
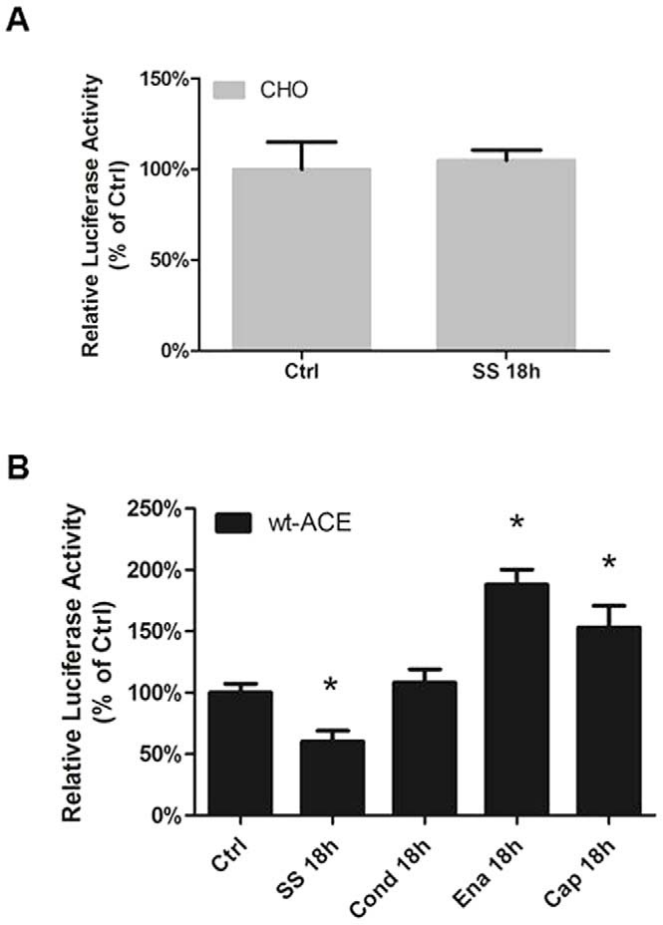
ACE expression on cell surface is necessary for SS-induced downregulation of ACE promoter activity. ACE promoter activity assessed in (A) CHO cells, (B) wt-ACE cells submitted to shear stress for 18 hours (15 dyne/cm^2^; SS 18 h). Effects of shear-conditioned medium for 18 h (Cond 18 h) and treatment of wt-ACE cell with Enalapril or Captopril (1 µM, 18 h) were also assessed in wt-ACE. The results are represented as relative luciferase activity of static control cells. Each bar is mean ± SEM of 5 to 7 separate experiments. *p<0.05 vs static control (CTRL).

Wild type CHO transiently tranfected with the 1,274 bp of the rat ACE promoter fused to a reporter gene displayed a significantly level of activity, which was not influenced by SS (18 h, 15 dyn/cm^2^) (Figure 4A). In contrast, there was a significant 40% decrease in promoter activity in wt-ACE CHO (Figure 4B, Static 100±7.2%; wt-ACE, 59.9±8.9%). These data provide additional evidence indicating that the cell model system used recapitulates the endogenous features of endothelial cells in which ACE expression is negatively influenced by SS (Figure 1A). Noteworthy, the expected up regulation of ACE promoter activity associated with the exposure to ACE inhibitors was observed (Figure 4B, 191±5% to Enalapril and 156±7% to Captopril).

**Figure 5:**
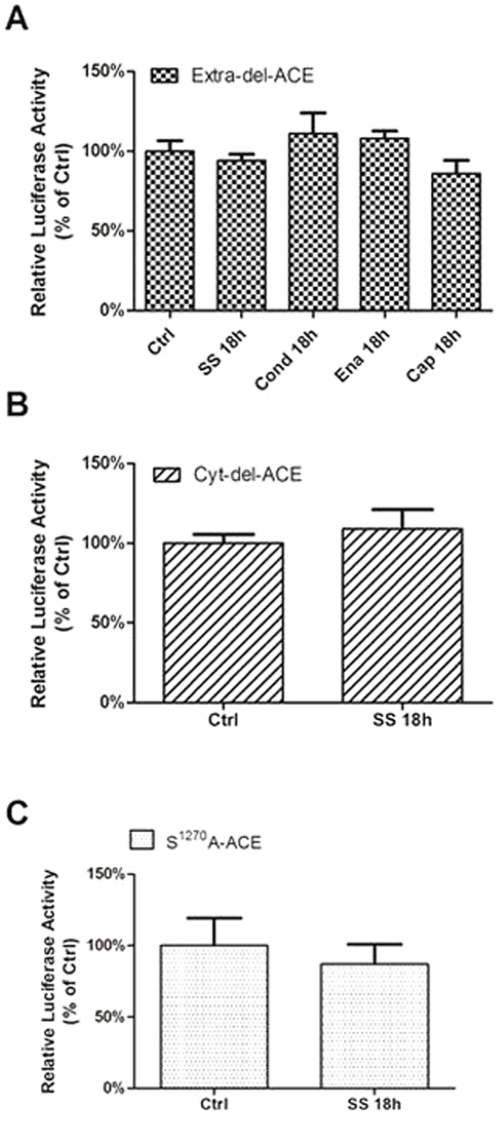
ACE extracellular and intracellular domains are necessary for SS-induced downregulation of ACE promoter activity. ACE promoter activity assessed in (A) Extra-del-ACE cells, (B) Cyto-del-ACE and (C) S^1270^A-ACE cells submitted to shear stress (15 dyne/cm^2^; SS 18 h). Effects of shear-conditioned medium (Conditioning for 18 h) and treatment of wt-ACE cell with Enalapril or Captopril (1 µM, 18 h) were also assessed in Extra-del-ACE cells. The results are represented as relative luciferase activity of static control cells. Each bar is mean ± SEM of 5 to 7 separate experiments. *p<0.05 vs static control (CTRL).

Then, ACE promoter activity was investigated in CHO cells permanently transfected with ACE lacking the intra- or the extracellular domains, or carrying a mutation at residue Ser^1270^, Cyt-del-ACE, Extra-del-ACE, and S^1270^A-ACE cells, respectively. Interestingly, the SS-mediated response on ACE promoter activity was abrogated in all cell lines (Figure 5A–C) suggesting that the extra- and the intracellular ACE domains as well as the Ser^1270^ are required for the SS-induced response in wt-ACE (Figure 4B). Note that ACE inhibitors treatment on Extra-del-ACE cells failed to induce activation of the ACE promoter activity as observed above (Figure 4B).

### ACE activation by shear stress occurs by direct effect of mechanical stimulus

To evaluate whether SS-mediated decrease in ACE promoter function is also influenced by the SS-induced release of autocrine/paracrine factors, both static wt-ACE and Extra-del-ACE were exposed to sheared-conditioned medium (18 h, 15 dyn/cm^2^). ACE promoter activity remained unchanged by the SS-conditioned medium (Figure 4B and 5A) suggesting that SS-induced ACE decreased expression is not secondary to the release of factor(s) stimulated by the mechanical force, which is in agreement with previous data from our group showing that NO does not modulate ACE under SS condition [13].

### JNK as a downstream inhibitory pathway associated with SS-induced ACE downregulation

**Figure 6:**
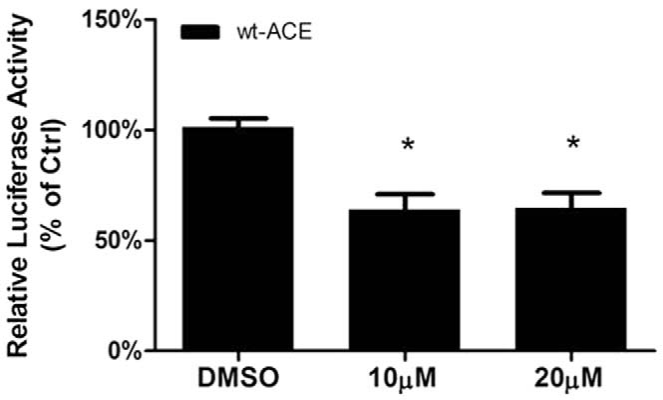
JNK inhibition decrease ACE promoter activity in wt-ACE cells. Cells were treated for 18 h with 10 µM or 20 µM SP600125. Each bar represents mean ± SEM of 4 to 6 separate experiments. *p<0.05 vs. static control (CTRL).

The role of decreased p-JNK in inhibiting ACE promoter function was tested by stimulating wt-ACE cells transfected with the ACE promoter reporter gene with the JNK inhibitor SP600125 in absence of SS. The JNK inhibitor decreased ACE promoter activity (control, 100±4.3%; 10 µM, 64±6.6%; 20 µM 64.7±6.7%) (Figure 6), indicating that a diminished JNK activation *per se*, in the absence of the mechanical stimulus, can mimic the SS-mediated downregulation of ACE expression. This finding provides no direct evidence for the role of JNK in this response, but the potential link between JNK pathway and ACE expression downregulation is underscored taking into consideration the data shown on figure 3 and 4.

### Shear stress-mediated down regulation of ACE counteracts the ACE inhibitor-mediated up regulation of ACE in endothelial cells

**Figure 7:**
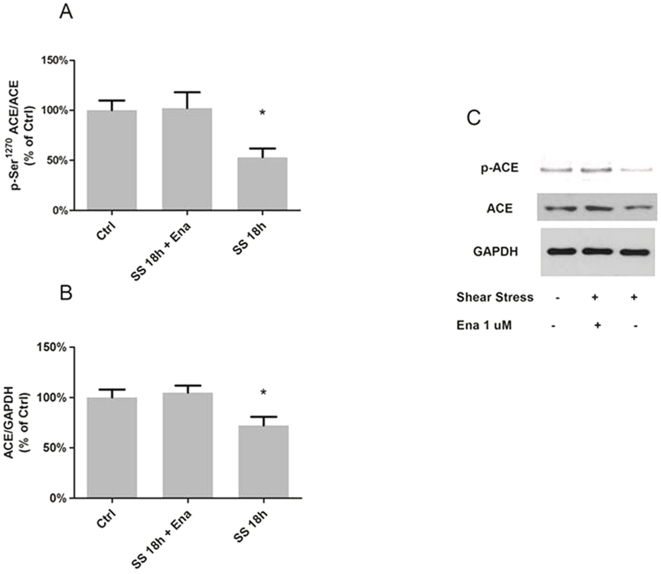
SS-induced decrease in ACE and phosphorylation on Ser^1270^ is counteracted by ACE inhibitor treatment. (A) ACE phosphorylation on Ser^1270^, (B) ACE protein expression downregulation, and (C) Representative western blots. Saphenous vein endothelial cells were concomitant submitted to laminar shear stress (15 dyne/cm^2^; SS 18 h) and treated with ACE inhibitor enelapril (Ena, 1 µM). Each bar represents mean ± SEM of 3 to 4 separate experiments. *p<0.05 vs control (CTRL).

Considering that the ACE inhibitor-mediated up regulation of ACE is associated with augmentation of p-ACE at Ser^1270^ and ACE expression [14] and that the SS-induced response has opposite effects, we verified if there is a prevailing influence when EC are exposed simultaneously to both conditions, as it may happens *in vivo* during ACE inhibition. Interestingly, the presence of both stimuli rendered the ratio of pACE/ACE as well as the ACE expression unchanged suggesting that the stimuli had opposing effects of similar magnitude on these responses (Figure 7A–C).

## Discussion

The results of the present study provide original evidence that SS-induced downregulation of ACE in endothelial cells is modulated by changes in the phosphorylation status of residue Ser^1270^ in the ACE cytoplasmic tail and downstream JNK pathway. Using a CHO cell model system that enabled the expression of wild type ACE or selected mutants that made the cells recapitulate key events of the natural response in endothelial cells, we were able to demonstrate that both ACE extra- and intracellular domains of the molecule as well as the S^1270^ residue are required for the mechanotransduction of SS. In regard to the effects on ACE phosphorylation, JNK activation, and ACE expression, the SS and ACEi appears to have opposing effects raising the possibility that blood flow pattern itself may affect the action of agents such as ACEi and deserves to be further explored in the future.

This is the first demonstration that a transmembrane enzyme behaves as a mechanosensor, and it amplify the scope of candidate molecules that can sense mechanical stimuli. Integrins have been considered the main candidates for shear sensors, but molecules including ion channels and VEGF receptor have also been demonstrated to be able to sense SS and initiate intracellular signal transduction [15]. More recently, the enzyme phospholipase A2 (PLA2) has also been suggested to be a mechanosensor, but the data were obtained using a very artificial system of lypophilic miscelae [16].

Recently, it has been demonstrated that the cytoplasmic tail of ACE is phosphorylated on Ser^1270^ (p-Ser^1270^) by ACE inhibitors (ACEi), which then triggers intracellular signaling cascade that leads to increase ACE expression [6]. The binding of ACEi to ACE induces p-Ser^1270^ mediated by casein kinase 2 activating MKK7 and JNK. Then, phosphorylated c-jun activates AP-1 transcription factor and increase ACE expression [7], [8], [9].

We previously demonstrated that SS decreases ACE expression and activity using a combination of *in vitro* and *in vivo* approaches [10]. More recently, Fleming's group showed that ACE can be phosphorylated at the intracellular domain upon ACEi binding to the molecule resulting in augmentation of ACE expression [2]. The idea that the large extracellular domain of ACE can behave as a mechanosensor is intriguing especially considering the privileged location of ACE in endothelial cells as well as in epithelia in renal tubules that are exposed to flow. Thus, we investigated whether ACE behaves as a mechanosensor and influences its downregulation in response to SS in primary culture of endothelias cells, whereas the CHO model system allowed precisely dissection of the response. The data with ACE mutants are consistent with the model where ACE can both sense and convert the SS stimulus in outside-in signaling involving changes in the phosphorylation status of Ser^1270^, downstream JNK inhibition and diminished ACE expression. Thus, SS modulates components of the same pathway described for ACEi, although in opposite direction as mentioned before.

The JNKs are traditionally considered stress-activated protein kinases involved in several cellular processes including cell proliferation, apoptosis, migration, cytoskeleton rearrangements, inflammation, metabolic disease, neurodegenerative disease, oncogenesis, and cancer progression [17], [18]. JNK are usually activated by inflammatory cytokines and environmental stresses, including UV irradiation, osmotic stress, redox stress, and mechanical stress [19]. The isoforms JNK1 and JNK2 are ubiquitously expressed, while JNK3 is mainly expressed in the heart, brain, and testis [20]. JNK activation induces the activator protein-1 transcription factor and increases the expression of inflammatory genes such as monocyte chemotactic protein-1 [21], interleukin-8 [22], and VCAM-1 [23]. Moreover, JNK by modulating cytokines and adhesion molecules can mediate leukocyte recruitment and activation and participates in the atherosclerotic process [24]. This is better demonstrated when inhibition or deleting JNK reduces atherosclerosis lesions in ApoE−/− mice in hypercholesterolemia-induced endothelial dysfunction and oxidative stress [25]. On the other hand, it has been described that JNK can also mediate apoptosis under stress conditions and thus could contribute to the elevated rates of apoptosis at atheroprone regions in vivo [20]. Our results are showing that SS decreases ACE phosphorylation and JNK activation, which may represent additional atheroprotective mechanisms of shear stress. This is consistent with the observation that atherosclerosis usually appears in regions of curvature or bifurcations where the flow is disturbed with low shear stress. We may also speculate that in these regions JNK and ACE are more active, since they are excluded from the SS-induced response, favoring atherosclerosis progression [26], [27].

The broad usage of ACEi in cardiovascular diseases is generally attributed to the conversion of Ang I and the accumulation of bradykinin, although the role of the latter is a matter of debate [28], [29]. The findings presented here raise the possibility that other properties may also influence the beneficial use of ACEi, especially in the targets where physical influences do play a role like the vascular system and the sites where atherosclerotic lesions are located and associated with disturbances in the blood flow pattern. Considering that ACEi activates JNK and ACE expression, that SS-induced response has the opposite effect, and that SS levels are not constant either in physiological or pathological conditions [30], it is tempting to speculate that the control of ACE levels under both conditions are variable and most importantly that the effects of an ACEi may vary according to local flow pattern.

We also investigated whether the SS-induced ACE signaling activation is directly a consequence of the mechanical force or secondary to SS-induced released factor(s). The sustained exposure of ECs to laminar shear stress is accompanied by the release of several antiatherogenic factors, including prostacyclin (PGI2), transforming growth factor-β (TGF- β) and nitric oxide (NO) [31]. We have previously demonstrated that NO is not involved in ACE downregulation by SS, although NO can regulate ACE under basal static conditions [12], [13]. Here, we used shear-conditioned medium and observed no modification on ACE promoter activity under the conditions tested. Even though this finding is consistent with a direct effect of the mechanical forces, It must be emphasized that there is a limitation in this approach since many of the candidate factors may be unstable, e.g. NO and Ang II, and may not be active in the conditioned medium for long periods. Together, the data suggest that ACE downregulation by SS occurs indeed by the direct effect of mechanical force in ACE phosphorylation since (i) wild-type ACE expression in the cell membrane is required for ACE downregulation, and (ii) SS-conditioned medium had no effect on ACE expression modulation.

Thus, the decrease of ACE signaling by shear stress might also contribute to reduce Ang II formation and Bradykinin accumulation and all its consequences on diminishing the oxidative stress and Ang II-induced inflammatory responses [32]. Furthermore, our findings highlight the fact that blood flow may affect the action of agents such as ACEi and, therefore, the proper control of blood flow should be an efficient therapeutic alternative in addition to the traditional interventions [33]. The ACE mechanotransdution downregulates both JNK and ACE activation, which may be considered SS-induced atheroprotective effects on the vascular wall. It will be important to assess whether similar phenomenum can be observed in other sites where cells expressing ACE are also submitted to changes in flow such as in the renal tubules.

The main claims of the present study were obtained using primary endothelial cells, although key aspects of the underlying molecular mechanisms were clarified using a versatile cell model system, which recapitulate the data observed with the endogenous system. An alternative to the use of CHO cell system, which lacks endogenous expression of ACE, is the use of endothelial cells lacking ACE from the ACE knockout mouse, however, these cells require the use of transient transfections since primary endothelial cells are not amenable to multiple passages required for the selection procedure. The transient transfection system is less robust than the permanently transfected cells expressing different ACE mutants. Thus, we believe the proposed strategy, despite its own limitations, was instrumental to give additional support to the main claims of the study.

Taken together, we provide the first evidence for an enzyme as a mechanosensor to shear stress. The data show that ACE influences its own regulation in response to SS and suggest that ACE extracellular domain behaves as a mechanosensor while the cytoplasmic domain elicits the downstream intracellular signaling by phosphorylation on Ser^1270^.

## Methods

### Cell Culture

Primary culture of endothelial cells was obtained as previously described [34]. Human saphenous vein were obtained from patients undergoing aortocoronary bypass surgery in the Heart Institute (InCor), University of São Paulo Medical School [34]. All individuals gave written informed consent to participate in the study, which was reviewed and approved by the local Ethics Committee (Comissao de Etica para Analise de Projetos de Pesquisa do Hospital das Clinicas da Faculdade de Medicina da Universidade de Sao Paulo, SDC 2454/04/074 – CAPPesq 638/04). Briefly, luminal surface of human saphenous vein was incubated with 1 mg/mL collagenase type II for 1 h at 37°C. The vessel was flushed with phosphate buffer solution and the cell pellet was cultured in Human Endothelial-SFM (Invitrogen) supplemented with 20% of new born calf serum, 20 ng/mL FGF, 10 ng/mL EGF, 10 U/mL penicillin, 10 mg/mL streptomycin, and 10 U/mL heparin. All the experiments were performed with cells up to 8th passage.

Chinese hamster ovary (CHO) cells were obtained from Sigma-Aldrich. Collection. CHO cells were grown in 100-mm culture dishes in Dulbeccos's modified Eagle Medium (DMEM) High Glucose culture medium supplemented with L-glutamine, antibiotic (penicillin and streptomycin), Hepes buffer, and 10% fetal bovine serum. Cells were routinely sub cultured using trypsin-EDTA to mobilize them. For transfection, CHO cells were plated in 60-mm dish, 1 day prior to transfection.

### Cellular Fractioning

Immediately following the experimental protocol, cell were lysed in ice-cold lysis buffer (20 mM Tris-HCl, pH 7.5, 2 mM EDTA, 10 mM EGTA, 0.25 M Sucrose, and 1∶300 protease and phosphatase inhibitors (Sigma)). The homogenate was centrifuged at 100 *g* for 5 min (4°C). The resulting supernatant was centrifuged again at 100.000 *g* for 30 min (4°C) to obtain the pellet (nucleus and cell membrane) and the cytoplasmic extract (supernatant). The pellet was incubated with the same lysis buffer containing 1% TritonX100 and DNAse (1 mg ml^−1^) for 30 min (4°C) and then centrifuged at 100.000 *g* for 30 min (4°C) to obtain the cell membrane (supernatant).

### Construction of CHO cells lineages expressing wild-type and individual domains of ACE

cDNAs of human wild-type ACE, Cyt-del-ACE, Extra-del-ACE and S^1270^A-ACE were kindly provided by Dr Pierre Corvol (INSERM, and Collège de France, Paris, France) [35]; Dr Ervin G Erdos (University of Illinois, Laboratory of Peptide Research, Chicago, USA) [36], Dr Nigel Hooper (University of Leeds, School of Biochemistry and Molecular Biology, Leeds, UK) [37] and Dr Ingrid Flemming (Johann Wolfgang Goethe University, Institute for Vascular Signaling, Frankfurt, Germany) [6] respectively. Those constructs were inserted into the pcDNA3 plasmid (carrying the neomycin resistance gene). CHO cells were stable transfected with ACE constructs using the Lipofectamin method and neomycin-resistant cells were selected with G418. Clones were isolated by cloning rings and grown to confluence. The success of all clone construction and selection was confirmed by mRNA and protein expression.

### Gene expression by RT-PCR

Total RNA was isolated with Trizol Reagent according to the manufacturer's instructions and cDNA synthesis was performed with radom hexamers (High Capacity cDNA Archive kit-PE Applied Biosystem). The reaction was carried out using Taq polymerase under the following conditions: initial denaturation for 5 min at 95°C followed by 40 cycles of denaturation for 15 s at 95°C, annealing for 1 min at 60°C, extension for 1 min at 72°C, and final extension for 10 min at 72°C. The PCR products were analysed by electrophoresis on agarose gel.

The primers used were: Primer 1 antisense 5′-ACCTCGGAGCCGAACTGGGG-3′, sense 5′-GGCTGCTGCTCTTCCTGGGC-3′ amplify the fragment between 3801 and 3924 in the ACE cytoplasmic domain; Primer 2 antisense 5′-TCCGGGATGTGGCCATCACA-3′, sense 5′-CCTGCCCAGGAGCTGGAGGA-3′ amplify the fragment between 2219 and 2357 in the ACE extracellular domain.

### Transient transfection with ACE promoter fused to luciferase reporter gene

CHO and CHO expressing wild-type ACE or ACE mutants were transfected with luciferase reporter plasmid (pGL2 vector, Promega) harboring the 1,274 bp of rat ACE promoter gene [10] by LIPOFECTAMIN method (Invitrogen). pRL-SV40 was co-transfected as an internal control following the manufacturer protocol. Luciferase and Renila activities were measured using the Dual-Luciferase Reporter Assay System (Promega Luciferase Assay System) using a luminometer (Monolight 2010, Analytical luminescence laboratory).

On figure 4b and 5a, “Ctrl” refer to the cells maintained under static condition (not stimulated by shear stress) and “Cond 18 h” refer to the cells treated with shear- conditioned media. After 18 h of shear stress, the media from the cells submitted to shear stress for 18 h was collected and added to a new plate of cells for another 18 h. It was performed to evaluate whether SS-mediated decrease in ACE promoter function is also influenced by the SS-induced release of autocrine/paracrine factors. This approach is an attempt to separate the effects of released factor(s) stimulated by shears stress and the direct mechanical influence over ACE molecule in the cell membrane.

### Shear stress protocol

Primary cultures of ECs and CHO cell lineages were submitted to controlled shear stress as previously described [11], [34], [38]. Cells were plated in 100 mm dishes pre-coated with gelatin 1% and before shearing the cells were serum starved for 24 hours. Shear stress at 15 dyne/cm^2^ was produced by a cone plate viscometer as described before [39]. At the end of the experiment, the cells were washed with cold phosphate buffered solution and lysed to further analysis.

### Western Blot analysis

Cells were lysed in lysis buffer (1 mM EDTA, 1 mM EGTA, 2 mM MgCl_2_, 5 mM KCl, 25 mM HEPES, 1 mM PMSF, 2 mM DTT, 0,1%Triton X-100 and protease inhibitor cocktail (Sigma-Aldrich). After 10 min on ice, samples were centrifuged at 10,000 g for 10 min to remove cellular debris. Cell lysates (5 to 40 µg) supernatant were heated in SDS-PAGE sample buffer, fractioned by SDS-PAGE and transferred to Hybond membranes (GE Healthcare). Transfer efficiency was monitored by 0.5% Ponceau S staining. The blotted membranes were first blocked with 5% non-fat milk for 2 h at room temperature and then proteins were detected using their respective antibodies. The p-JNK and JNK antibodies were obtained from CellSignaling (1∶1000), the p-Ser^1270^ ACE antibody (1∶1000) was provided by Dr Ingrid Fleming from the Goethe University, Frankfurt, Germany; the Caveolin-1 (1∶1000, Sta. Cruz), the ERK1/2 (1∶1000, CellSignaling), and the ACE antibody by Dr Sergei M Danilov from the University of Illinois at Chicago, USA. Horseradish peroxidase-conjugated antibody was used as a secondary antibody, and signals were detected using the ECL detection kit (GE Healthcare).

### Statistical Analysis

All data are representative of at least 3 independent experiments. Numerical data era presented as mean±SEM. Comparisons among the groups were performed with student *t-*test or 1-way ANOVA and appropriate posthoc Tukey comparison. Statistical significance was accepted if p<0.05.

## References

[1] Bader M, Ganten D (2008). Update on tissue renin-angiotensin systems.. J Mol Med.

[2] Fleming I, Kohlstedt K, Busse R (2005). New fACEs to the renin-angiotensin system.. Physiology (Bethesda).

[3] Coates D (2003). The angiotensin converting enzyme (ACE).. Int J Biochem Cell Biol.

[4] Resnick N, Yahav H, Shay-Salit A, Shushy M, Schubert S (2003). Fluid shear stress and the vascular endothelium: for better and for worse.. Prog Biophys Mol Biol.

[5] Chien S (2007). Mechanotransduction and endothelial cell homeostasis: the wisdom of the cell.. Am J Physiol Heart Circ Physiol.

[6] Fleming I (2006). Signaling by the angiotensin-converting enzyme.. Circ Res.

[7] Kohlstedt K, Shoghi F, Muller-Esterl W, Busse R, Fleming I (2002). CK2 phosphorylates the angiotensin-converting enzyme and regulates its retention in the endothelial cell plasma membrane.. Circ Res.

[8] Kohlstedt K, Brandes RP, Muller-Esterl W, Busse R, Fleming I (2004). Angiotensin-converting enzyme is involved in outside-in signaling in endothelial cells.. Circ Res.

[9] Kohlstedt K, Busse R, Fleming I (2005). Signaling via the angiotensin-converting enzyme enhances the expression of cyclooxygenase-2 in endothelial cells.. Hypertension.

[10] Rieder MJ, Carmona R, Krieger JE, Pritchard KA, Greene AS (1997). Suppression of angiotensin-converting enzyme expression and activity by shear stress.. Circ Res.

[11] Miyakawa AA, de Lourdes Junqueira M, Krieger JE (2004). Identification of two novel shear stress responsive elements in rat angiotensin I converting enzyme promoter.. Physiol Genomics.

[12] Ackermann A, Fernandez-Alfonso MS, Sanchez de Rojas R, Ortega T, Paul M (1998). Modulation of angiotensin-converting enzyme by nitric oxide.. Br J Pharmacol.

[13] Pertrini CM, Miyakawa AA, Laurindo FR, Krieger JE (2003). Nitric oxide regulates angiotensin-I converting enzyme under static conditions but not under shear stress.. Braz J Med Biol Res.

[14] Ryan MJ, Sigmund CD (2004). ACE, ACE inhibitors, and other JNK.. Circ Res.

[15] Hahn C, Schwartz MA (2009). Mechanotransduction in vascular physiology and atherogenesis.. Nat Rev Mol Cell Biol.

[16] Lehtonen JY, Kinnunen PK (1995). Phospholipase A2 as a mechanosensor.. Biophys J.

[17] Johnson GL, Nakamura K (2007). The c-jun kinase/stress-activated pathway: regulation, function and role in human disease.. Biochim Biophys Acta.

[18] Kyriakis JM, Avruch J (2001). Mammalian mitogen-activated protein kinase signal transduction pathways activated by stress and inflammation.. Physiol Rev.

[19] Raman M, Chen W, Cobb MH (2007). Differential regulation and properties of MAPKs.. Oncogene.

[20] Davis RJ (2000). Signal transduction by the JNK group of MAP kinases.. Cell.

[21] Chien S (2006). Molecular basis of rheological modulation of endothelial functions: importance of stress direction.. Biorheology.

[22] Cheng M, Wu J, Li Y, Nie Y, Chen H (2008). Activation of MAPK participates in low shear stress-induced IL-8 gene expression in endothelial cells.. Clin Biomech (Bristol, Avon).

[23] Verna L, Ganda C, Stemerman MB (2006). In vivo low-density lipoprotein exposure induces intercellular adhesion molecule-1 and vascular cell adhesion molecule-1 correlated with activator protein-1 expression.. Arterioscler Thromb Vasc Biol.

[24] Sumara G, Belwal M, Ricci R (2005). “Jnking” atherosclerosis.. Cell Mol Life Sci.

[25] Osto E, Matter CM, Kouroedov A, Malinski T, Bachschmid M (2008). c-Jun N-terminal kinase 2 deficiency protects against hypercholesterolemia-induced endothelial dysfunction and oxidative stress.. Circulation.

[26] Hahn C, Orr AW, Sanders JM, Jhaveri KA, Schwartz MA (2009). The subendothelial extracellular matrix modulates JNK activation by flow.. Circ Res.

[27] Fukuhara M, Geary RL, Diz DI, Gallagher PE, Wilson JA (2000). Angiotensin-converting enzyme expression in human carotid artery atherosclerosis.. Hypertension.

[28] Vanhoutte PM, Boulanger CM, Illiano SC, Nagao T, Vidal M (1993). Endothelium-dependent effects of converting-enzyme inhibitors.. J Cardiovasc Pharmacol.

[29] Dandona P, Dhindsa S, Ghanim H, Chaudhuri A (2007). Angiotensin II and inflammation: the effect of angiotensin-converting enzyme inhibition and angiotensin II receptor blockade.. J Hum Hypertens.

[30] Cheng C, Helderman F, Tempel D, Segers D, Hierck B (2007). Large variations in absolute wall shear stress levels within one species and between species.. Atherosclerosis.

[31] Traub O, Berk BC (1998). Laminar shear stress: mechanisms by which endothelial cells transduce an atheroprotective force.. Arterioscler Thromb Vasc Biol.

[32] Dzau VJ (2001). Theodore Cooper Lecture: Tissue angiotensin and pathobiology of vascular disease: a unifying hypothesis.. Hypertension.

[33] Richter Y, Edelman ER (2006). Cardiology is flow.. Circulation.

[34] Campos LC, Miyakawa AA, Barauna VG, Cardoso L, Borin TF (2009). Induction of CRP3/MLP expression during vein arterialization is dependent on stretch rather than shear stress.. Cardiovasc Res.

[35] Soubrier F, Alhenc-Gelas F, Hubert C, Allegrini J, John M (1988). Two putative active centers in human angiotensin I-converting enzyme revealed by molecular cloning.. Proc Natl Acad Sci U S A.

[36] Marcic B, Deddish PA, Skidgel RA, Erdos EG, Minshall RD (2000). Replacement of the transmembrane anchor in angiotensin I-converting enzyme (ACE) with a glycosylphosphatidylinositol tail affects activation of the B2 bradykinin receptor by ACE inhibitors.. J Biol Chem.

[37] Pang S, Chubb AJ, Schwager SL, Ehlers MR, Sturrock ED (2001). Roles of the juxtamembrane and extracellular domains of angiotensin-converting enzyme in ectodomain shedding.. Biochem J.

[38] Bassaneze V, Barauna VG, Ramos CL, Kalil JE, Schettert IT (2009). Shear Stress Induces Nitric Oxide-mediated VEGF Production in Human Adipose Tissue Mesenchymal Stem Cells.. Stem Cells Dev.

[39] Malek A, Izumo S (1992). Physiological fluid shear stress causes downregulation of endothelin-1 mRNA in bovine aortic endothelium.. Am J Physiol.

